# Supplementary material for: Indexed variation graphs for efficient and accurate resistome profiling
Source: Bioinformatics. 2018 May 14;34(21):3601–8. doi: 10.1093/bioinformatics/bty387 (PMC6198860; doi:10.1093/bioinformatics/bty387)

# Indexed variation graphs for efficient and accurate resistome profiling

Will P. M. Rowe *

will.rowe@stfc.ac.uk

Institute of Integrative Biology, The University of Liverpool

† Scientific Computing Department and The Hartree Centre, STFC Daresbury Laboratory

Martyn D. Winn

martyn.winn@stfc.ac.uk

Scientific Computing Department and The Hartree Centre, STFC Daresbury Laboratory

† current address

* corresponding author

## Supplementary Methods

**Algorithm 1:** Indexing a variation graph

*G* ← topologically sorted variation graph

X ← initalised LSH forest index

L ← number of buckets in X

H ← empty array of initial hash tables

B ← empty array of hashed signature buckets and the associated graph locations

**for all** traversals *t* in *G* **do**

**for all** windows *w* in *t* **do**

*w_sig_* ← getMinHash(*w*) // *w_sig_* is the window MinHash signature

*w_loc_* ← getLocation(*t, w*) // w_loc_ locates *w_sig_* in the graph

**for all** buckets *b* in 1 *..* L **do**

*w*_lsh_ ← lsh(*w_sig_, b*) // *w_lsh_* is a hashed chunk of *w_sig_*

**if** *w_lsh_* ∈ H[*b*] **then**

H[*b*][*w_lsh_*] ← H[*b*][*w_lsh_*] + *w_loc_*

**if** *w_lsh_* ∉ H[*b*] **then**

H[*b*][*w_lsh_*] ← *w_loc_*

**for all** buckets *b* in 1 .. L **do**

**for all** *w_lsh_* and locations *l* in H[*b*] **do**

add *w_lsh_* and *l* to B

sort(B)

X[*b*] ← B // copy B to LSH forest index

clear B

remove H[*b*]

**return** X

**Algorithm 2:** Tuning the LSH Forest

L ← number of buckets to use in the LSH Forest

K ← number of hash functions to use in the LSH Forest

E ← minimum error found

S ← length of MinHash signature

J ← Jaccard similarity threshold

P ← precision

L, K ← 0, 0 // start with L and K zeroed

E ← MaxFloat64 // set E to a maximum value

**for all** *buckets b* in 1 .. S **do**

**for all** *functions f* in 1 .. S **do**

**if** (*b* * *f*) > S **then**

**break**

*fp* ← fPos(*b*, *f*) // calculate the false positive rate

*fn* ← fNeg(*b*, *f*) // calculate the false negative rate

*e* ← *fp* + *fn*

**if** *e* < E **then**

E ← *e* // set the minimum error to the current error

L ← *b*

K ← *f*

**return** L, K

**function** fPos(b, f)

pdf ← probabilityDensityFunction(b, f)

prob_fPos ← 0.0

**for all** *values v* in 0 .. J **do**

prob_fPos += pdf(*v* + 0.5*P) * P

*v* += P

**return prob_fPos**

**end function**

**function** fNeg(b, f)

pdf ← probabilityDensityFunction(b, f)

prob_fNeg ← 0.0

**for all** *values v* in J .. 1.0 **do**

prob_fPos += pdf(*v* + 0.5*P) * P

*v* += P

**return prob_fNeg**

**end function**

**Algorithm 3:** Querying a set of indexed variation graphs

X ← LSH forest index of 1 or more variation graphs

L ← number of buckets in X

P ← size of prefix used to compress internal nodes of X

Q ← a MinHash signature for a query FASTQ read

A ← empty array to store query results

**for all** buckets *b* in 1 *..* L **do**

*c* ← lsh(Q, *b*) // a chunk of the query is hashed to a binary string

*v* ← X[*b*][:P] // the current LSH bucket is compressed with a prefix

*m* ← search(*c*, *v*) // binary search to find matching LSH branches

**for all** branches *r* in *m* **do**

**if** *c* == *r* **then**

**for all** keys *k* in *r* **do**

**if** *k* not in A **then**

add *k* to A

**return** A

**Algorithm 4:** Aligning a read to a variation graph

N ← node in variation graph

R ← a seeded FASTQ read

P ← alignment path of read against variation graph

L ← length of alignment path

**function** ALIGN (N, R, P, L)

**if** N == R[L] **then** // compare the node against the current read base

add N to P // add the node to the alignment path

increment L

**else**

**return** P

**if** L == length(R) **then** // report path if the all bases in the read match

**return** P

**for all** edges *e* in N **do**

N ← *e*

ALIGN (N, R, P, L)

**end function**

## Supplementary Results

### Supplementary Results 1.

Table showing the genes annotated by GROOT and AMRPlusPlus from the synthetic dataset (spiked with genes from MegaRes).

| **Tool** | **Threshold (% reference covered)** | **Number of correctly annotated genes** | **Number of false positive annotations** | **Number of false negative annotations** |
| --- | --- | --- | --- | --- |
| GROOT | 100 | 10 | 4 | 0 |
|  | 99 | 10 | 8 | 0 |
|  | 80 | 10 | 93 | 0 |
| AMRPlusPlus | 100 | 1 | 11 | 9 |
|  | 99 | 9 | 12 | 1 |
|  | 80 | 10 | 58 | 0 |

List of the spiked genes from the MegaRes database (entries are headers taken from the MegaRes database FASTA file).

1. 270|M33768.1|BACBMRX|Multi-drug_resistance|Multi-drug_efflux_pumps|BMR
2. AGly|EU886977.1|gene2|Aminoglycosides|Aminoglycoside_N-acetyltransferases|AAC6-PRIME
3. Flq|NC_003098.1.934295|Fluoroquinolones|Fluoroquinolone-resistant_DNA_topoisomerases|PARC|RequiresSNPConfirmation
4. Sul|NC_010410.6003232|Sulfonamides|Sulfonamide-resistant_dihydropteroate_synthases|FOLP|RequiresSNPConfirmation
5. Bla|IMP-21|AB204557|1-738|738|betalactams|Class_B_betalactamases|IMP
6. Bla|TEM-112|AY589493|167-1027|861|betalactams|Class_A_betalactamases|TEM
7. Flq|NC_002695.1.916822|Fluoroquinolones|Fluoroquinolone-resistant_DNA_topoisomerases|GYRA|RequiresSNPConfirmation
8. gi|698174217|gb|KM087863.1|betalactams|Class_C_betalactamases|MIR
9. gi|90992729|gb|DQ445683.1|betalactams|Class_D_betalactamases|OXA
10. CARD|phgb|X82668|187-922|ARO:3000250|ErmC|MLS|23S_rRNA_methyltransferases|ERMC

### Supplementary Figure 1.

This figure shows a boxplot comparing the toxin-classified reads from all rural vs. urban subject microbiomes.


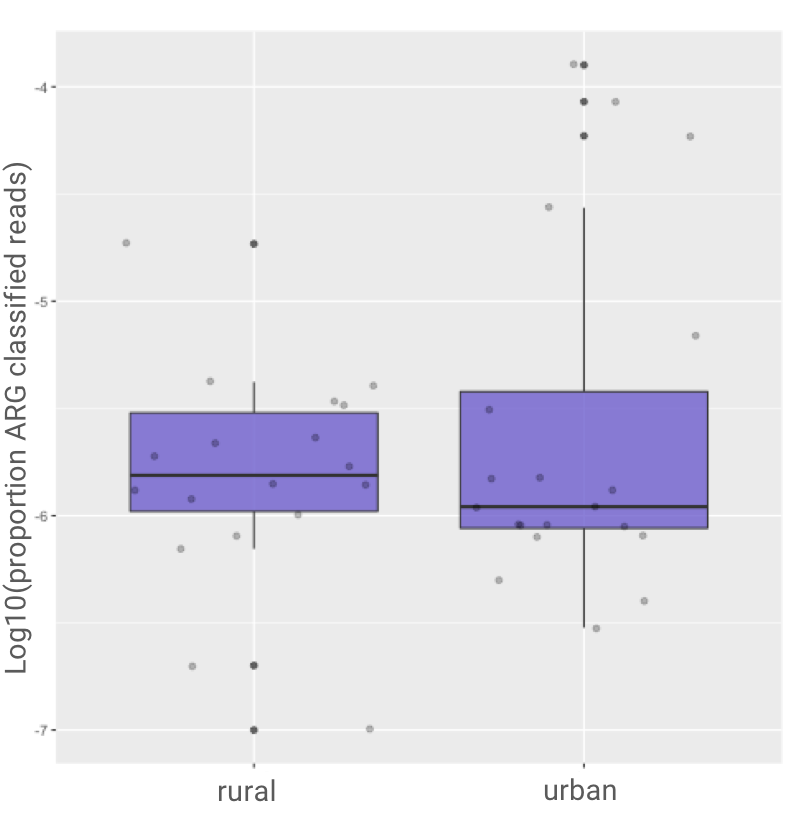

Supplement: Supplementary Material [file bty387_supplementary_material.docx]
